# Four-year review of safe and effective procedural sedation in neonates and young infants

**DOI:** 10.3389/fphar.2024.1381413

**Published:** 2024-07-26

**Authors:** Yu Cui, Min Tang, Qixia Mu, Qunying Wu, Lu Kang, Qin Chen, Yani He

**Affiliations:** Department of Anesthesiology, The Affiliated Hospital, School of Medicine, UESTC Chengdu Women’s and Children’s Central Hospital, Chengdu, China

**Keywords:** neonates, infants, chloral hydrate, midazolam, procedural sedation

## Abstract

**Objectives:**

Newborns and small infants are unable to cooperate actively during diagnostic procedures; therefore, sedation is often employee to maintain immobilization and obtain high-quality images. However, these procedures are often indicated in sick, vulnerable, or hemodynamically unstable neonates and young infants, which raises the associated risks of sedation. This study summarizes our 4-year of experience with safe and effective procedural sedation in this vulnerable population.

**Study design:**

This retrospective study analyzed data on neonates and young infants who underwent non-painful diagnostic procedures from December 2019 to November 2023. Patients were categorized into the neonate (aged≦ 28 days) and the young infant (29 days ≦ aged ≦ 90 days) groups.

**Results:**

Non-pharmacological strategies, including sleeping naturally, swaddling/facilitated tucking, non-nutritive sucking, and skin-to-skin care, can achieve a success rate for sedation about 98.4%. In terms of pharmacological methods, our institution primarily utilizes chloral hydrate for procedural sedation in neonates and young infants undergoing non-painful diagnostic procedures. Midazolam serves as an alternative sedative. Chloral hydrate alone demonstrated a 92.5% success rate on the first attempt, compared to midazolam alone, with an 85.11% success rate. Neonates experienced a higher incidence of adverse events during sedation compared to young infants.

**Conclusion:**

This study reviews our 4-year experience with procedural sedation in neonates and young infants. Chloral hydrate demonstrated a high degree of safety and efficacy in this population. However, supervision by skilled medical personnel and extended observation is required. In our institution, the experience with midazolam is limited in this population, and further research is warranted to establish its safety and efficacy. Non-pharmacological strategies can achieve an acceptable rate of sedation success, which can be used based on patient’s tolerance.

## Background

Newborns and small infants are inherently unable to cooperate actively during diagnostic procedures. To ensure minimal movement and obtain high-quality images during non-painful diagnostic procedures, such as magnetic resonance imaging (MRI), computed tomography (CT), or echocardiography, sedation is often necessary. Nonpharmacologic strategies have garnered significant attention; however, their widespread adoption remains limited due to the lack of standardization protocols for specific procedures, inconsistent application durations, and a dearth of longitudinal studies (McPherson and Grunau, 2022).

In recent years, various sedatives have recently become available. However, the administration of these sedatives in neonates and young infants requires caution as the therapeutic window between sedation and anesthesia is very narrow (Havidich et al., 2016). Moreover, neonates or young infants requiring diagnostic procedures are sick and vulnerable, or even hemodynamically unstable, which may increase the risk of sedation. Any adverse event occurring during the process can potentially lead to parental dissatisfaction.

Furthermore, the paucity of literature regarding the efficacy and safety of sedatives in neonates and young infants often prompts off-label use. For these providers may be reluctant to conduct sedation for newborns and young infants. Our institution is a large tertiary women’s and children’s hospital in southwest of China, with over 300 neonatal intensive care units (NICU) beds. More than 3,000 neonates and young infant undergo non-painful diagnostic procedural sedation annually. Thus, we summarize our 4-year experience of safe and effective procedural sedation in neonates and young infants.

## Materials and methods

This retrospective study analyzed data on neonates and young infants who underwent non-painful diagnostic procedures at Chengdu Women’s and Children’s Hospital sedation center from December 2019 to November 2023. Ethical approval was obtained from the institutional board of Chengdu Women’s and Children’s Central Hospital [No. 2024 (2)]. Written informed consent was waived due to the anonymity of the participants. The study was conducted in accordance with the Declaration of Helsinki.

### Patients selection

Patients were selected through electronic medical records. The inclusion criteria were as follows: ① Only neonates (aged ≦ 28 days) and small infants (29 days ≦ aged ≦ 90 days) were enrolled in the current study; ② Patients who underwent non-painful diagnostic procedures. Our institution restricted referrals to the sedation center to non-invasive or painless sedation only; whereas cases requiring sedation for painful procedures were handled solely by the anesthesiology department, as detailed in our previous work (Cui et al., 2023). Non-painful diagnostic procedures in this study encompassed non-invasive procedures such as magnetic resonance imaging (MRI), computed tomography (CT), echocardiography, lung function testing, hearing screening, and visual and auditory evoked potentials (VAEPs). Patients requiring assisted ventilation and those with incomplete data were excluded from the analysis.

### Data collection and outcome measures

The retrospective data, including age (days), gender, weight, admission status (inpatients/outpatients), procedural type, sedated methods (non-pharmacological or pharmacological), details of the initial sedative (type, dose, route of administration), sedation failure with the initial dose, need for rescue medication, sedation success with the initial dose, sedation duration, and complications. Non-pharmacological methods encompass diagnostic procedures completed without medications. These included sleeping naturally, swaddling/facilitated tucking, non-nutritive sucking, and skin-to-skin care. Sedation failure with the initial dose was defined as the patients could not complete the procedure with the initial sedative(s). Sedation duration was referred to as the time from administration of sedatives to patient discharge from the sedation center. The analysis also documented any complications, including vomiting, bradycardia, agitation, delayed awakening, desaturation, and respiratory depression. Vomiting is defined as the patient spitting out medication or stomach contents after administration. Bradycardia is defined as a heart rate below 100 bpm (Fleming et al., 2011; Estkowski et al., 2015). Delayed awake is defined as a sedation duration exceeding 120 min. Respiratory depression is airway obstruction with oxygen saturation (SpO_2_) ≥90% or respiratory rates <8 times per minute. Desaturation referred to SpO_2_ < 90% for more than 10 s.

Patients were categorized into two groups: the neonate (aged ≦ 28 days) and the young infant (29 days ≦ aged ≦ 90 days) group.

### Statistical analysis

Continues variables are presented as mean ± standard deviation if normally distributed, and Student’s t-tests were used for comparison between group comparisons. Non-normally distributed variables were presented as median (Q1–Q3), and the Mann-Whitney U test was used for comparisons. The distribution of the variables was evaluated by the Shapiro normality test when the sample size <5,000, otherwise Kolmogorov-Smirnov test should be used. The categorical variables are expressed as count (percentage). The Fisher exact test or χ^2^ was used as appropriate. A significance level of 0.05 was considered significant. Analyses were conducted by R studio, version 4.2.2.

## Results

A total of 13,517 neonates and young infants underwent diagnostic procedural sedation during the study period. Non-pharmacological strategies were employed for sedation in 245 (1.8%) patients, including 188 neonates and 57 infants. Compared to infants, more neonates accepted non-pharmacological ways to complete the procedures. About 98.4% (241/245) of patients achieved the targeted sedation depth and completed the procedure solely by non-pharmacological methods. Details of the sedation characteristics are provided in Supplementary Table S1.

**FIGURE 1:**
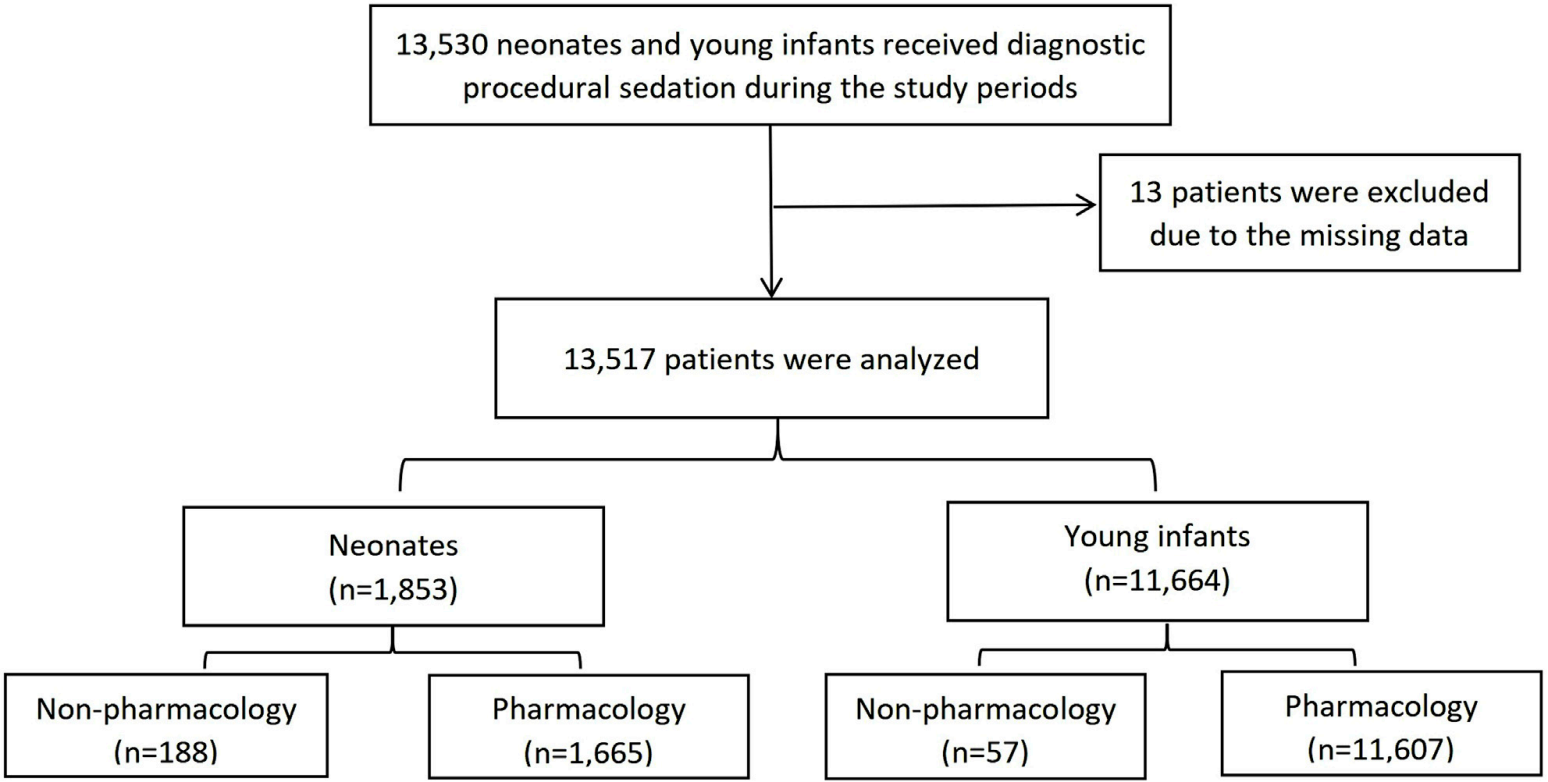
The study flow chart.

**FIGURE 2:**
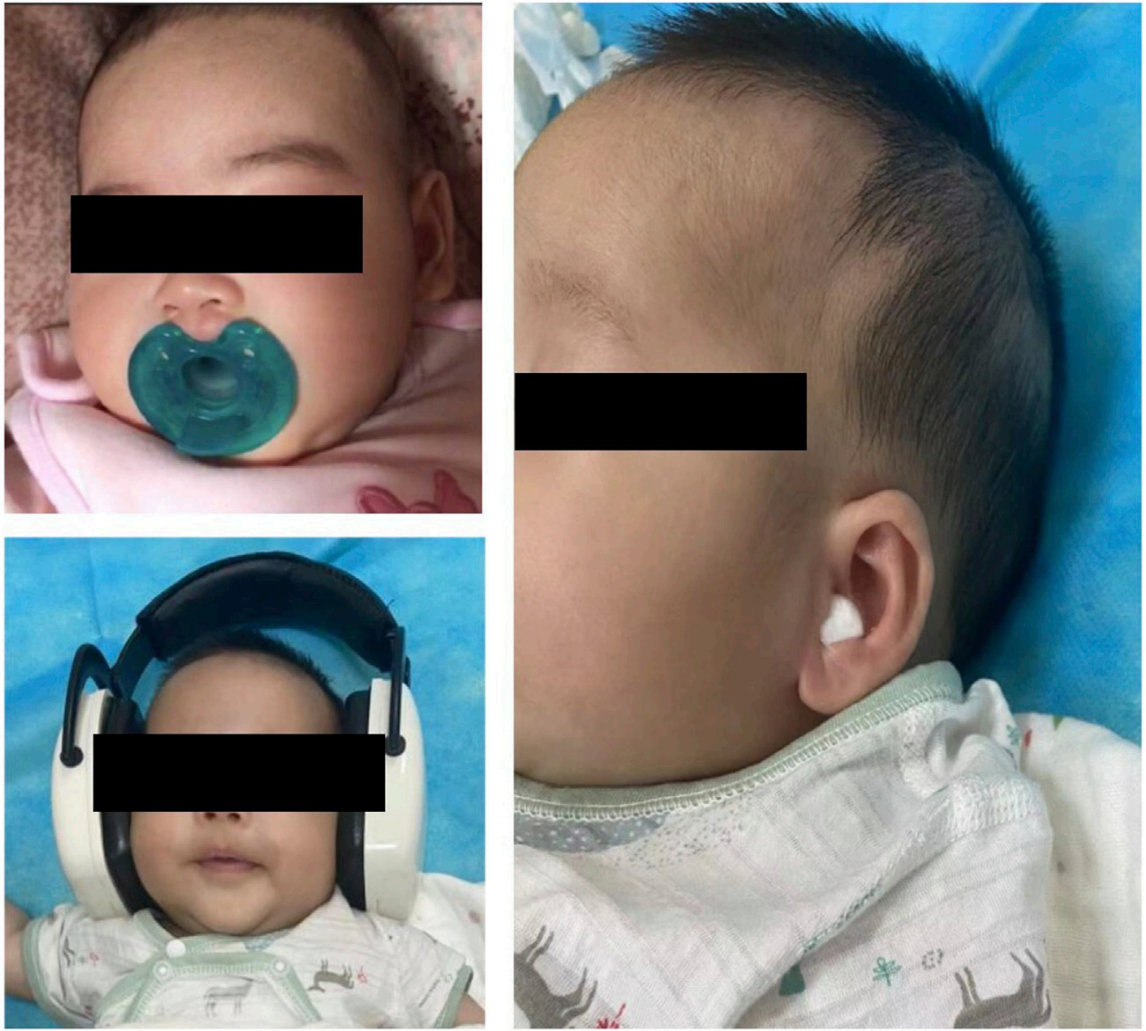
Nonpharmacological strategies during procedural sedation, including non- and nutritive sucking, pacifier, earplugs, and noise-canceling headphones.

Among pharmacological strategies, sedative was administered to 1,665 neonates and 11,607 infants. The study flow chart is shown in Figure 1. Demographics are presented in Table 1. The most frequent non-painful diagnostic procedures requiring sedation in neonates and young infants were hearing screening (n = 4,842, 35.8%), MRI (n = 3,252, 24.1%), lung function tests (n = 2,337, 17.3%), echocardiography (n = 1861, 13.8%), CT scans (n = 407, 3.0%), visual and auditory evoked potential (VAEP) (n = 356, 2.6%) and MRI + VAEP (n = 259, 1.9%) (Table 1). Nonpharmacological strategies during procedural sedation, including nutritive and non-nutritive sucking, pacifiers, earplugs, and noise-canceling headphones, are shown in Figure 2.

**TABLE 1:** Characteristics of study subjects, procedures performed.

Characteristics	All patients (n = 13,517)	Neonates (n = 1853)	Young infants (n = 11,664)
Age, days, median (IQR)	59 (37, 71)	11 (6, 16)	60 (49, 75)
Weight, kg, median (IQR)	5.0 (4.2, 5.8)	3.3 (2.9, 3.7)	5.2 (4.6, 6.0)
Gender, males, n (%)	7,835 (58.0)	1,118 (60.4)	6,717 (57.6)
Type of patients			
Inpatients, n (%)	5,647 (41.8)	1,496 (80.7)	4,151 (35.6)
Outpatients, n (%)	7,870 (58.2)	357 (19.3)	7,513 (64.4)
Sedation history (yes), n(%)	1,692 (12.5)	221 (11.9)	1,471 (12.6)
Procedures, n (%)			
Hearing screening	4,842 (35.8)	8 (0.4)	4,834 (41.4)
MRI	3,252 (24.1)	1,470 (79.3)	1782 (15.3)
Lung function tests	2,337 (17.3)	8 (0.4)	2,329 (20.0)
Echocardiography	1861 (13.8)	8 (0.4)	1853 (15.9)
CT	407 (3.0)	53 (2.9)	354 (3.0)
VAEP	356 (2.6)	96 (5.2)	260 (2.2)
MRI + VAEP	259 (1.9)	205 (11.1)	54 (0.5)
CT + Lung function test	44 (0.3)	0 (0.0)	44 (0.4)
Lung function test + Echocardiography	54 (0.4)	0 (0.0)	54 (0.5)
MRI + Echocardiography	40 (0.3)	1 (0.1)	39 (0.3)
CT + Echocardiography	18 (0.1)	1 (0.1)	17 (0.1)
MRI + CT	9 (0.1)	0 (0.0)	9 (0.1)
MRI + Lung function tests	8 (0.1)	0 (0.0)	8 (0.1)
Others	6 (0.0)	1 (0.1)	5 (0.0)
Hearing screening + VAEP	5 (0.0)	0 (0.0)	5 (0.0)
Lung function tests + VAEP	5 (0.0)	1 (0.1)	4 (0.0)
MRI + Echocardiography + VAEP	3 (0.0)	0 (0.0)	3 (0.0)
MRI + Hearing screening	2 (0.0)	1 (0.1)	1 (0.0)
Echocardiography + VAEP	3 (0.0)	0 (0.0)	3 (0.0)
CT + Lung function tests + VAEP	1 (0.0)	0 (0.0)	1 (0.0)
CT + ECG	1 (0.0)	0 (0.0)	1 (0.0)
MRI + Lung function tests + Echocardiography	1 (0.0)	0 (0.0)	1 (0.0)
MRI + Others	1 (0.0)	0 (0.0)	1 (0.0)
Non-pharmacological stratigies, n(%)	245 (1.81)	188 (10.15)	57 (0.49)
Pharmacological stratigies, n(%)			
Chloral hydrate single used	13,204 (97.68)	1,635 (88.24)	11,569 (99.19)
Chloral hydrate + Midazolam	4 (0.03)	1 (0.05)	3 (0.03)
Chloral hydrate + Dexmedetomidine	8 (0.06)	0 (0.00)	8 (0.07)
Chloral hydrate + Propofol	1 (0.01)	1 (0.05)	0 (0.00)
Midazolam single used	47 (0.35)	24 (1.30)	23 (0.20)
Midazolam + Dexmedetomidine	2 (0.01)	0 (0.00)	2 (0.02)
Midazolam + Propofol	1 (0.01)	1 (0.05)	0 (0.00)
Propofol	3 (0.02)	2 (0.11)	1 (0.01)
Dexmedetomidine	2 (0.01)	1 (0.05)	1 (0.01)

Note: Computed tomography (CT); Electrocardiography (ECG); Magnetic resonance imaging (MRI); Visual and auditory evoked potential (VAEP).

### Sedatives

Of the 13,272 patients who underwent pharmacological procedural sedation, almost all (n = 13,256, 99.9%) received a single sedative agent. Multiple sedatives were administered in only 16 (0.1%) of the cases. Chloral hydrate was the most frequently administered initial sedative (n = 13,217, 99.6%). It served as the sole sedative in 13,204 cases and was combined with propofol (1 case), dexmedetomidine (8 cases), and midazolam (4 cases). This indicates a strong preference for chloral hydrate as the primary sedative for both neonates and small infants in our institution. Midazolam was used as the sole sedative in only 47 cases (0.3%), and was combined with propofol (1 case), dexmedetomidine (2 cases), and chloral hydrate (4 cases). Dexmedetomidine was used in 2 cases as the sole sedative, and propofol was used alone in only 3 cases.

#### Chloral hydrate

Table 2 details the specific use of chloral hydrate for diagnostic procedural sedation. The initial dose across the entire cohort was 50.0 mg/kg (IQR, 49.9, 50.0). Neonates required a lower dose of 49.4 mg/kg (IQR: 48.1, 50.0) compared to young infants who received a dose of 50.0 mg/kg (IQR: 49.1, 50.0) (*p* < 0.01) to maintain adequate depth of sedation during the diagnostic procedures. Chloral hydrate was administered orally, rectally, or via gastric tubes. The preferred initial administration route for chloral hydrate was orally, but some patients received the medication via gastric tube. A significantly higher proportion of neonates (n = 126, 7.71%) required gastric tube administration compared to small infants (23, 0.20%) (*p* < 0.01), suggesting more frequent pre-procedural tube placement in neonates than in young infants. The initial chloral hydrate dose achieved successful sedation in 90.9% of neonates, which was lower compared to young infants (92.7%). Rescue sedatives included additional chloral hydrate, dexmedetomidine, midazolam, and propofol. Compared to young infants, sedation duration was shorter in the neonate group.

**TABLE 2:** The detailed strategies used for diagnostic procedural sedation with chloral hydrate.

Chloral hydrate	All patients (n = 13,217)	Neonates (n = 1,637)	Young infants (n = 11,580)	*P* values
Chloral hydrate single used	13,204 (99.9)	1,635 (99.9)	11,569 (99.9)	
Initial dose used, median (IQR),mg/kg	50.0 (49.9, 50.0)	49.4 (48.1, 50.0)	50.0 (49.1, 50.0)	<0.01*
Initial administration route, n (%)				<0.01*
Orally	12,879 (97.5)	1,489 (91.1)	11,390 (98.5)	
Rectal	165 (1.25)	19 (1.16)	146 (1.26)	
Gastric tube	149 (1.13)	126 (7.71)	23 (0.20)	
Unknown	11 (0.08)	1 (0.06)	10 (0.09)	
Initial success rate, n (%)	12,216 (92.5)	1,487 (90.9)	10,729 (92.7)	0.01*
Sedation duration, median (IQR),min	62.2 (49.0, 83.2)	58.2 (47.0, 76.1)	63.1 (49.1, 84.3)	<0.01*
	(n = 11,973)	(n = 1,599)	(n = 10,374)	
Adverse events, n (%)				<0.01*
Agitation	2 (0.02)	1 (0.01)	1 (0.01)	
Aspiration	1 (0.00)	0 (0.00)	1 (0.01)	
Bradycardia	1 (0.00)	1 (0.01)	0 (0.00)	
Cough	1 (0.00)	0 (0.00)	1 (0.01)	
Desaturation	18 (0.14)	11 (0.12)	7 (0.06)	
Desaturation + vomiting	1 (0.00)	0 (0.00)	1 (0.01)	
Desaturation + bradycardia	1 (0.00)	0 (0.00)	1 (0.01)	
Respiratory depression	4 (0.03)	1 (0.01)	4 (0.03)	
Vomiting	173 (1.31)	76 (0.84)	97 (0.84)	
Chloral hydrate combined used	13 (0.10)	2 (0.12)	11 (0.10)	NA
Chloral hydrate + Midazolam	4 (0.03)	1 (0.06)	3 (0.03)	
Chloral hydrate + Dexmedetomidine	8 (0.06)	0 (0.00)	8 (0.07)	
Chloral hydrate + Propofol	1 (0.01)	1 (0.06)	0 (0.00)	
Initial success rate, n (%)	13 (100.0)	2 (100.0)	11 (100.0)	NA
Sedation duration, median (IQR), min	64.1 (53.43, 67.27)	63.1 ± 0.1	68.0 ± 28.2	<0.01*
	(n = 10)	(n = 2)	(n = 8)	
Adverse events, n (%)	0 (0.00)	0 (0.00)	0 (0.00)	NA

Note: Intravenous injection (IV); Interquartile range (IQR).

#### Midazolam

Midazolam was used alone in 47 neonates and young infants during procedure sedation, with 24 cases in the neonate group and 23 in the young infant group. Neither group had any statistically significant differences in the initial dose, administration route, success rate, and sedation duration (Table 3). The rescue medications for midazolam sedation were chloral hydrate, dexmedetomidine, and midazolam.

**TABLE 3:** The detailed strategies used for diagnostic procedural sedation with midazolam.

Midazolam	All patients (n = 54)	Neonates (n = 26)	Young infants (n = 28)	
Midazolam single used, n (%)	47 (87.04)	24 (92.31)	23 (82.14)	*P* values
Initial dose used, median (IQR), mg/kg	0.49 (0.29, 0.50)	0.49 (0.48, 0.50)	0.40 (0.26, 0.49)	0.15
Initial administration route, n (%)				0.58
Orally	34 (72.34)	19 (79.17)	15 (65.22)	
IV	7 (14.89)	3 (12.50)	4 (17.39)	
Intranasal	6 (12.77)	2 (8.33)	4 (17.39)	
Initial success rate, n (%)	40 (85.11)	22 (91.67)	18 (78.3)	0.24
Sedation duration, mean ± SD, min	46.7 ± 19.3	44.0 ± 15.5	49.7 ± 23.4	0.34
Adverse events				0.49
Bradycardia	1 (1.85)	0 (0.00)	1 (4.35)	
Midazolam combined used, n (%)	7 (12.96)	2 (7.69)	5 (17.86)	0.33
Midazolam + Chloral hydrate	4 (7.41)	1 (3.85)	3 (10.71)	
Midazolam + Dexmedetomidine	2 (3.70)	0 (0.00)	2 (7.14)	
Midazolam + Propofol	1 (1.85)	1 (3.85)	0 (0.00)	

Note: IQR (Interquartile range); IV (Intravenous); SD (Standard deviation).

### Adverse events

Procedural sedation resulted in adverse events in 206 (1.52%) of the 13,517 cases (Table 1). The incidence was significantly higher in neonates (n = 92, 4.96%) than in young infants (n = 114, 0.98%) (*p* < 0.01). Vomiting was the most frequent adverse event affecting 1.28% of the entire cohort, followed by desaturation (n = 18, 0.13%) and respiration depression (n = 5, 0.04%). The rate of desaturation in the entire cohort was 0.13%, with 0.59% in the neonate group and 0.06% in the young infant group. Desaturation was well alleviated in all patients by oxygen therapy, posture changes, and mask ventilation. Respiration depression occurred in 5 cases (1 neonate and 4 small infants) during procedural sedation, and all cases were improved by pain stimulation or jaw lift/chin tilt. Two young infants experienced more than one side effect—desaturation + bradycardia and desaturation + vomiting. No severe adverse events, such as cardiac arrest or death, were reported. Patients who required repeat dosing due to sedation failure with the initial dose experienced more adverse events (OR 76.3; 95% CI 52.1, 114.4) (Table 4).

**TABLE 4:** Analysis of adverse events according to the efficacy of sedation.

Adverse events	All patients	Sedation success with initial dose	Sedation failure with initial dose	*OR, 95%CI*
	(n = 13,517)	(n = 12,517)	(n = 1,000)	*P* value
None, n(%)	13,311 (98.5)	12,483 (99.7)	828 (82.8)	76.3 (52.1, 114.4) <0.01*
Complication distribution, n(%)				
Vomiting	173 (1.3)	11 (0.3)	162 (16.2)	
Rash	0 (0.00)	0 (0.00)	0 (0.00)	
Agitation	2 (0.01)	1 (0.03)	1 (0.10)	
Aspiration	1 (0.01)	1 (0.03)	0 (0.00)	
Bradycardia	4 (0.03)	4 (0.12)	0 (0.01)	
Cough, n (%)	1 (0.01)	0 (0.00)	1 (0.00)	
Respiratory depression, n (%)	5 (0.04)	5 (0.15)	0 (0.00)	
Desaturation, n (%)	18 (0.13)	11 (0.3)	7 (0.70)	
Desaturation and bradycardia, n (%)	1 (0.01)	0 (0.00)	1 (0.10)	
Desaturation and vomiting, n (%)	1 (0.01)	1 (0.03)	0 (0.00)	
Cardiac arrest or death, n (%)	0 (0.00)	0 (0.00)	0 (0.00)	

Note: *P value < 0.05.

## Discussion

The study summarized a 4-year experience of procedural sedation in neonates and young infants undergoing non-painful diagnostic procedures. Chloral hydrate was the most preferred sedative for neonates and young infants. Midazolam was also employed for procedural sedation. The success rate for chloral hydrate alone was 92.5% (first attempt), compared to midazolam alone (85.11%). Neonates experienced a higher incidence of adverse events compared to those reported in young infants during procedural sedation.

### Non-pharmacological strategies

A multi-national survey from nine different countries reported that 96% of participating sites used non-pharmacological sedative practices in infants under 6 months old (Greer et al., 2024). However, the success rate associated with these non-pharmacological strategies varied substantially. Heller et al. (2017) evaluated neonatal brain MRI procedures in NICUs throughout the United States using a 15-question survey. Of the 96 programs surveyed, 58 responded, and 64% used non-pharmacological strategies, such as feed and swaddle. In the group using non-pharmacological strategies, 81% reported a failure rate of <25% in obtaining useful images; 11% reported a failure rate of 25%–75%; and 5% reported a failure rate >75% (Heller et al., 2017).

In our study, we found that non-pharmacological strategies were employed only in 1.81% (245/13,517) of the cases. Of these, 76.7% (188/245) were neonates, and 23.3% (57/245) were young infants. Thus, non-pharmacological strategies had low adoption rates. The possible explanations were as follows: ① The non-pharmacological strategies included swaddling, pacifiers, and wearing noise-canceling headphones. Although feeding is a non-pharmacological strategy to promote sedation success (Windram et al., 2012; Eker et al., 2017), we rarely use it. This is because if useful images are not obtained, rescue sedatives may be prescribed, which require at least a 2-h fast before the medication is administered (Joshi et al., 2023). ② In a high-volume institution like ours, the time it takes to prepare patients can decrease productivity. Additionally, a potential reduction in diagnostic accuracy may prompt more parents to opt for sedation. ③ Newborn’s naturally sleep for about 16 h (Sadeh et al., 1996), and most newborns who arrive at the sedation center are in their natural sleep phase. Therefore, sedative providers may recommend initial non-pharmacological methods approaches. However, during the first 6 months, as they get older, nocturnal sleep is extended and becomes more consolidated, while daytime sleep decreases (Sades and Sivan, 2009). Young infants often arrive at the sedation center awake or crying, prompting the selection of pharmacological methods to avoid repeated attempts. Thus, compared to infants, more neonates accepted non-pharmacological ways to complete the procedures. The rate of sedation success through non-pharmacological strategies was high (about 98.4%) when patients prepared very well. The choice of non-pharmacological strategies, however, relies on the patient’s tolerance.

### Pharmacological strategies

#### Chloral hydrate

Several recent studies have reported the use of chloral hydrate (Finnemore et al., 2014; Cui et al., 2022), midazolam (Inserra et al., 2022), and dexmedetomidine (Inserra et al., 2022; Wang et al., 2024) were used for procedural sedation in neonates and infants. Chloral hydrate single used was the most preferred drug for procedural sedation in neonates and young infants at our institution, accounting for 97.68% (13,204/13,517) of cases. In 2023, a meta-analysis had well-summarized pharmacological sedation techniques in pediatric patients. Of the 67 studies included, chloral hydrate was used alone in 33 studies (2,883 cases), with combined use in 6 studies (Wang et al., 2024). In some countries, such as France, and Italy, chloral hydrate was not commercially available as a sedative due to its concerns about its side effect, including delayed awaking, hyperactivity, and nervousness (de Rover et al., 2023). However, it is still used in the developing countries (Fazli et al., 2023). The direct evidence of side effects related to chloral hydrate remains unconfirmed. One previous review suggested that chloral hydrate use required the supervision of skilled medical personnel and extended observation (Coté et al., 2000). Procedural sedation in our institution was performed by senior anesthesiologists possessing extensive clinical experience in managing adverse events, such as respiratory depression, airway obstruction, and desaturation. To prioritize patient safety, a dedicated team was assembled daily, comprising one anesthesiologist and six trained sedation nurses to oversee procedural sedation. To date, no severe adverse events occurred at the sedation center.

Previous studies have reported varying initial success rate for chloral hydrate sedation, ranging from 37.4% to 100% (de Rover et al., 2023). In our study, the initial success rate for the 13,204 cases who received chloral hydrate alone was 92.5%, with 90.9% in the neonate group and 92.7% in the young infant group (*p* < 0.01). Theoretically, the sedation success rate is influenced by the sedative dose and the characteristics of procedures. Longer and more invasive procedures, such as MRI examinations for multiple body parts (Cui et al., 2022), and painful procedures, are typically associated with higher failure rates. Conversely, non-painful procedures, such as those included in our study, have higher success rates, which may explain the high success rates observed in our cohort.

#### Midazolam

Midazolam also has a success rate ranging from 0% to 62% when used alone. However, midazolam may not be suitable for longer procedures (de Rover et al., 2023). In our study, the success rate for midazolam was 85.11% in the entire cohort, with 91.67% in neonates and 78.3% in young infants. This observed variability might be related to the differences in the study population. Our investigation specifically enrolled neonates and young infants, whereas previous studies included older children in their cohort. Older children had lower sleep requirements than infants, making it more difficult to achieve the desired sedation depth.

#### Dexmedetomidine

Dexmedetomidine, a highly selective α_2_-adrenergic receptor agonist, has gained widespread adoption for procedural sedation (Cui et al., 2023; Zhou et al., 2023). However, in our hospital, dexmedetomidine was rarely used for young infants due to concerns regarding bradycardia. A retrospective study comprising children (aged > 3 months) receiving intranasal dexmedetomidine for procedural sedation found that more than 2% of the children developed bradycardia (Lei et al., 2020). For young babies, their cardiac output is very sensitive to changes in heart rate. Episodes of bradycardia can lead to hypotension, asystole, and even death. Previously, we reported a neonate who experienced severe bradycardia during MRI sedation (Cui et al., 2020).

## Limitations

There are several limitations in this study. First, as a retrospective study, and incomplete data cannot be avoided. In our previous study, more than 3,000 records were excluded due to incomplete data (Finnemore et al., 2014). Thus, in 2023, to mitigate this limitation, our team members attempted to complete the data by scouring the medical records. And the quality of data has been improved greatly. Second, although all the data was collected from a large tertiary women’s and children’s hospital, which might provide valuable insights into our institution’s specific sedation practices and outcomes. However, the generalizability of these findings to other centers may be limited. The selection of procedural sedation agents may be limited by regional policies, drug availability, staff adequacy, and providers’ experience. Besides, the current study did not considered potential confounding factors that could impact sedation outcomes. These include underlying medical conditions, concomitant medications, or procedural complexity. Notably, this was a retrospective study, and most patients were outpatients, and these confounding factors were not well documented. Moreover, only neonates and young infants who underwent non-painful diagnostic procedures, and this specific patient population may not represent all neonates and young infants who require sedation for various procedures. For example, deep sedation and even anesthesia are required for painful procedures. However, our large sample size provides sufficient evidence. Next, the sedation score was not documented; thus, we could not analyze the depth of sedation for the enrolled patients. Due to the high volume of procedures at our sedation center, providers may not evaluate the sedation depth for each patient. However, successful completion of the diagnostic procedures indicates a satisfactory sedation depth. Last, our study population included preterm and full-term infants. It is well-established that preterm infants may exhibit differential responses to sedation compared to full-term infants. These variations can be attributed to their physiological immaturity and potential underlying medical conditions. An important area for future investigation is the safety and efficacy of sedation in both preterm and full-term infants and the influence of the specific sedative used, the dose administered, the patient’s overall health status, and underlying medical conditions. However, our retrospective design limited the ability to distinguish preterm neonates and full-term infants. Only the age (from the day of birth to the sedation day) was recorded. Future high-quality prospective cohort studies are required to elucidate the difference.

## Conclusion

In conclusion, this study summarizes 4 years of procedural sedation experiences in neonates and young infants, highlighting chloral hydrate as a safe and effective option for this patient population. However, supervision by skilled medical personnel and extended observation is required. The experience with midazolam use in our institution. Future studies are required to further explore its safety and efficacy. Additionally, our data indicate that non-pharmacological strategies can achieve an acceptable sedation success rate based on the patient’s tolerance.

## Data Availability

The raw data supporting the conclusions of this article will be made available by the authors, without undue reservation.

## References

[B1] Coté C. J. Karl H. W. Notterman D. A. Weinberg J. A. McCloskey C. (2000). Adverse sedation events in pediatrics: analysis of medications used for sedation. Pediatrics 106, 633–644. 10.1542/peds.106.4.633 11015502

[B2] Cui Y. Chen F. Xiao X. Li H. (2020). The delayed respiratory depression after dexmedetomidine sedation. J. Clin. Anesth. 65, 109886. 10.1016/j.jclinane.2020.109886 32464479

[B3] Cui Y. Gong T. Mu Q. Wu Q. Kang L. Chen Q. (2023). Predictors of pediatric sedation failure with initial dose of intranasal dexmedetomidine and oral midazolam. Pediatr. Res. 94, 2054–2061. 10.1038/s41390-023-02758-0 37507474

[B4] Cui Y. Guo L. Mu Q. Kang L. Chen Q. Wu Q. (2022). Analysis of risk factors for chloral hydrate sedative failure with initial dose in pediatric patients: a retrospective analysis. Paediatr. Drugs 24, 403–412. 10.1007/s40272-022-00511-4 35596111

[B5] de Rover I. Wylleman J. Dogger J. J. Bramer W. M. Hoeks S. E. de Graaff J. C. (2023). Needle-free pharmacological sedation techniques in paediatric patients for imaging procedures: a systematic review and meta-analysis. Br. J. Anaesth. 130 (1), 51–73. 10.1016/j.bja.2022.09.007 36283870

[B6] Eker H. E. Cok O. Y. Çetinkaya B. Aribogan A. (2017). Oral 30% glucose provides sufficient sedation in newborns during MRI. J. Anesth. 31, 206–211. 10.1007/s00540-016-2296-9 27999970

[B7] Estkowski L. M. Morris J. L. Sinclair E. A. (2015). Characterization of dexmedetomidine dosing and safety in neonates and infants. J. Pediatr. Pharmacol. Ther. 20, 112–118. 10.5863/1551-6776-20.2.112 25964728 PMC4418678

[B8] Fazli B. Hosseini S. A. Behnampour N. Langari A. Habibi-Koolaee M. (2023). Melatonin versus chloral hydrate on sleep induction for recording electroencephalography in children: a randomized clinical trial. Ann. Med. Surg. (Lond). 85, 5478–5483. 10.1097/MS9.0000000000001140 37915677 PMC10617919

[B9] Finnemore A. Toulmin H. Merchant N. Arichi T. Tusor N. Cox D. (2014). Chloral hydrate sedation for magnetic resonance imaging in newborn infants. Paediatr. Anaesth. 24, 190–195. 10.1111/pan.12264 24387147

[B10] Fleming S. Thompson M. Stevens R. Heneghan C. Plüddemann A. Maconochie I. (2011). Normal ranges of heart rate and respiratory rate in children from birth to 18 years of age: a systematic review of observational studies. Lancet 377, 1011–1018. 10.1016/S0140-6736(10)62226-X 21411136 PMC3789232

[B11] Greer M. C. Gee M. S. Pace E. Sotardi S. Morin C. E. Chavhan G. B. (2024). A survey of non-sedate practices when acquiring pediatric magnetic resonance imaging examinations. Pediatr. Radiol. 54, 239–249. 10.1007/s00247-023-05828-x 38112762

[B12] Havidich J. E. Beach M. Dierdorf S. F. Onega T. Suresh G. Cravero J. P. (2016). Preterm versus term children: analysis of sedation/anesthesia adverse events and longitudinal risk. Pediatrics 137, e20150463. 10.1542/peds.2015-0463 26917674 PMC9923625

[B13] Heller B. J. Yudkowitz F. S. Lipson S. (2017). Can we reduce anesthesia exposure? Neonatal brain MRI: swaddling vs. sedation, a national survey. J. Clin. Anesth. 38, 119–122. 10.1016/j.jclinane.2017.01.034 28372649

[B14] Inserra E. Colella U. Caredda E. Diplomatico M. Puzone S. Moschella S. (2022). Safety and effectiveness of intranasal dexmedetomidine together with midazolam for sedation in neonatal MRI. Paediatr. Anaesth. 32, 79–81. 10.1111/pan.14307 34618386 PMC9292475

[B15] Joshi G. P. Abdelmalak B. B. Weigel W. A. Harbell M. W. Kuo C. I. Soriano S. G. (2023). 2023 American society of anesthesiologists practice guidelines for preoperative fasting: carbohydrate-containing clear liquids with or without protein, chewing gum, and pediatric fasting duration-A modular update of the 2017 American society of anesthesiologists practice guidelines for preoperative fasting. Anesthesiology 138, 132–151. 10.1097/ALN.0000000000004381 36629465

[B16] Lei H. Chao L. Miao T. Ya Jun L. Shen Ling L. Yan Ying P. (2020). Incidence and risk factors of bradycardia in pediatric patients undergoing intranasal dexmedetomidine sedation. Acta Anaesthesiol. Scand. 64, 464–471. 10.1111/aas.13509 31736052

[B17] McPherson C. Grunau R. E. (2022). Pharmacologic analgesia and sedation in neonates. Clin. Perinatol. 49, 243–265. 10.1016/j.clp.2021.11.014 35210004

[B18] Sadeh A. Dark I. Vohr B. R. (1996). Newborns' sleep-wake patterns: the role of maternal, delivery and infant factors. Early Hum. Dev. 44, 113–126. 10.1016/0378-3782(95)01698-8 8745423

[B19] Sades A. Sivan Y. (2009). Clinical practice: sleep problems during infancy. Eur. J. Pediatr. 168, 1159–1164. 10.1007/s00431-009-0982-4 19343361

[B20] Wang X. Ma L. Yang X. Zhou Y. Zhang X. Han F. (2024). Efficacy of intranasal administration of dexmedetomidine in combination with midazolam for sedation in infant with cleft lip and palate undergoing CT scan: a randomized controlled trial. BMC Anesthesiol. 24, 10. 10.1186/s12871-023-02397-2 38166622 PMC10759416

[B21] Windram J. Grosse-Wortmann L. Shariat M. Greer M. L. Crawford M. W. Yoo S. J. (2012). Cardiovascular MRI without sedation or general anesthesia using a feed-and-sleep technique in neonates and infants. Pediatr. Radiol. 42, 183–187. 10.1007/s00247-011-2219-8 21861089

[B22] Zhou X. Zhao J. Tu H. Chen K. Hu Y. Jin Y. (2023). The effect of age on outpatient pediatric procedural sedation with intranasal dexmedetomidine and oral midazolam. Eur. J. Pediatr. 183, 169–177. 10.1007/s00431-023-05240-5 37855928 PMC10858144

