# Supplementary material for: Four-year review of safe and effective procedural sedation in neonates and young infants
Source: Front Pharmacol. 2024 Jul 26;15:1381413. doi: 10.3389/fphar.2024.1381413 (PMC11310124; doi:10.3389/fphar.2024.1381413)
Supplement: Supplementary file 1 [file Table1.docx]

**Supplementary table 1** Characteristics of patients underwent procedural sedation through non-pharmacological strategies

| **Characteristics** | **All patients (n=245)** | **Neonates (n=188)** | **Young infants (n=57)** |
| --- | --- | --- | --- |
| **Age, days, median (IQR)** | 15 (7, 27) | 10 (6, 17) | 40 (30, 57) |
| **Weight, kg, median (IQR)** | 2.8 (2.1, 3.4) | 3.0 (2.3, 3.4) | 2.1 (1.9, 2.5) |
| **Gender, males, n (%)** | 133 (54.3) | 105 (55.9) | 28 (49.1) |
| **Type of patients** |  |  |  |
| Inpatients, n (%) | 243 (99.2) | 187 (99.5) | 56 (98.2) |
| Outpatients, n (%) | 2 (0.8) | 1 (0.5) | 1 (1.8) |
| **Sedation history (yes), n(%)** | 31 (12.7) | 16 (8.5) | 15 (26.3) |
| **Procedures, n (%)** |  |  |  |
| MRI | 244 (99.6) | 187 (99.5) | 57 (100.0) |
| CT | 1 (0.4) | 1 (0.5) | 0 (0.0) |
| **Sedation success rate, n(%)** | 241 (98.4) | 186 (98.9) | 55 (96.5) |
| **Complication, n(%)** |  |  |  |
| Bradycardia | 1 (0.4) | 1 (0.5) | 0 (0.0) |

**Note**: Computed tomography (CT); Electrocardiography (ECG); Magnetic resonance imaging (MRI); Visual and auditory evoked potential (VAEP)
